# Spondylodiscitis: A Diagnostic and Management Dilemma

**DOI:** 10.7759/cureus.58284

**Published:** 2024-04-15

**Authors:** Akhshay J George, Srinivasalu Santhanagopal, Madan M Mohan, Jaya V Lal, Mallikarjunaswamy Basappa, Johann C Thomas, Jerin Jeevo

**Affiliations:** 1 Orthopaedics, St. Johns Medical College Hospital, Bangalore, IND

**Keywords:** pott's disease, tuberculous spondylitis, spine diseases, histopathology & microbiology, pyogenic, spondylodiscitis

## Abstract

Aims

Spondylodiscitis (SpD), a debilitating infective condition of the spine, mandates early diagnosis and institution of appropriate therapy, for which accurate microbiology and histological evaluation of the affected tissue is vital. The objectives of the study were to assess the correlation between clinical and magnetic resonance imaging (MRI) findings with histopathology (HPE) and microbiology (MB) in clinically diagnosed spondylodiscitis.

Settings and design

This was a prospective study of 34 consecutive patients reporting at the outpatient department of a tertiary hospital with clinical and imaging features of SpD, who underwent image-guided/surgical biopsy of lesions.

Methods and material

The provisional diagnosis of SpD in all patients was made on the combined basis of clinical profile and MRI Spine findings. Tissue samples in all patients, obtained by either open surgery or CT-guided biopsy, were subjected to HPE and MB analysis.

Results

SpD has a bimodal age distribution with the majority of patients being males in the fourth to fifth decades. Only raised erythrocyte sedimentation rate (ESR) was consistently seen amongst laboratory parameters, with leucocytosis being added pointer towards pyogenic etiology. MRI remained the imaging modality of choice for SpD but was not dependable for etiologic differentiation. On HPE and MB evaluations, 24 patients (71%) had findings consistent with infective SpD, while combined results augmented etiologic confirmation for 28 patients (82.4%). HPE was more sensitive than traditional MB methods to determine etiology in SpD, but the addition of the GeneXpert (Cepheid, Sunnyvale, California, United States) technique improved the MB positivity rate, especially in patients with tubercular SpD. Six patients (17.6%) with both negative HPE and MB results were categorized as 'Non-specific' SpD.

Conclusions

SpD poses a challenge to determine the etiology for the administration of specific antimicrobial therapy. A stratified standard institutional approach needs adoption to systematically evaluate SpD patients by having a high index of clinical suspicion, early imaging, followed by tissue biopsy for HPE and MB. Despite efforts to reach a diagnosis, a subset of patients without conclusive etiologic agent identification would remain as ‘Non-specific’, needing empiric antibiotic treatment based on clinico-radiologic profile.

## Introduction

Spondylodiscitis (SpD) is a combination of intervertebral disc infection with osteomyelitis of the juxta-discal regions of the vertebral bodies. The precise identification of the causative agent facilitates the timely institution of the correct therapeutic regimen and determines the need for orthopaedic intervention [1]. The prevalence of tuberculosis and pyogenic causative organisms is common in developing nations, but in recent times the list of causative organisms has significantly expanded the spectrum, including even fungi [2].

SpD diagnosis is clinically challenging due to its universally non-specific clinical presentation, and a targeted diagnostic approach is often based on a high index of clinical suspicion. Delays in diagnosis and treatment can result in significant morbidity in the form of restricted spinal mobility and neurological deficits. Magnetic resonance imaging (MRI) has now emerged as the most useful imaging modality to depict SpD but has not proved reliable in the accurate differentiation of etiologic agents [3]. Therefore, it becomes important to establish the definitive etiology based on the evaluation of tissue obtained from the site of suspected infection, in order to determine the optimal line of medical and orthopaedic management [4]. The present study was designed to evaluate the current role of histopathology (HPE) and microbiology (MB) in the diagnosis of SpD.

## Materials and methods

This was a prospective study of 34 consecutive adult patients presenting to the Outpatient Department of a Tertiary Care hospital, St. Johns Medical College Hospital, Bangalore, Karnataka, India, between September 2019 and August 2021. The study was approved by the Institutional Ethics Committee, St. Johns Medical College Hospital (approval number: 304/2019) for issues related to patient safety and confidentiality.

A provisional diagnosis of SpD in all patients was made on the combined basis of clinical profile and MRI Spine findings. Tissue samples in all patients, obtained by either open surgery or CT-guided biopsy, were subjected to HPE and MB analysis. HPE was done for ascertaining cytology and type of inflammatory response while MB tests included staining (Gram’s stain and Ziehl Neelsen stain), tissue culture for bacterial, fungal, and tuberculosis infections, and Xpert MTB/RIF assay (GeneXpert; Cepheid, Sunnyvale, California, United States), a highly sensitive molecular test for acid-fast bacilli (AFB).

HPE and MB results were tabulated and analyzed using IBM SPSS Statistics for Windows, Version 27.0 (Released 2020; IBM Corp., Armonk, New York, United States). For management facilitation, patients were classified as either pyogenic/tuberculosis SpD, if either or both HPE/MB tests were positive. Patients in whom both HPE and MB evaluation results proved inconclusive were placed under the subset of ‘Non-Specific’ SpD.

## Results

The study had a total of 34 participants, which included 19 male and 15 female patients. Ages ranged from 18 to 78 years (average age of 46 years). The most involved spinal level was lumbosacral in 50% (n=17) of the patients, followed by dorsal and dorsolumbar in 41% of patients (n=14), and the least in the cervical spine, involved in 8% of cases (n=3). ESR was raised in 91% of cases, while leucocytosis and/or elevated C-reactive protein (CRP) were seen only in 17% of the study population. The technique of tissue sampling was open biopsy (tissue sample collected from disease site during surgical intervention) in 29 patients and CT-guided biopsy in five subjects.

HPE and MB results 

**Figure 1:**
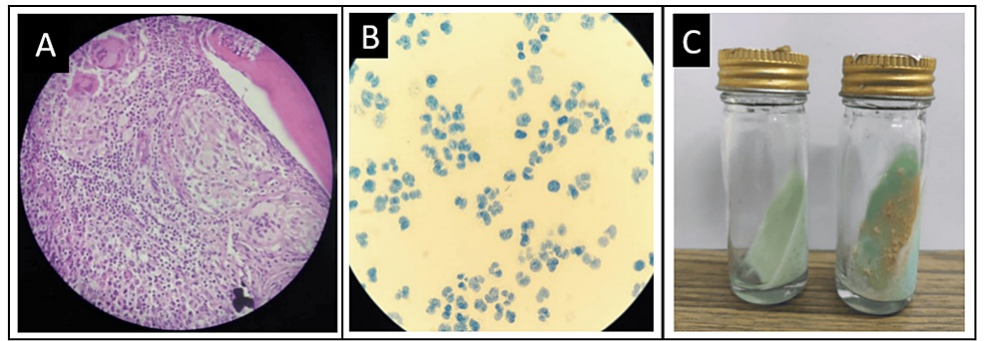
Tubercular spondylodiscitis (A) Pedicular biopsy dorsal spine (10 X) showing fibro-connective tissue stroma with granuloma composed of scattered epitheloid cells collection, abundant lymphocytes, and multiple Langhans type giant cells; features consistent with necrotizing granulomatous inflammation, likely of tubercular etiology. (B) Light microscopy image showing acid-fast bacilli on Ziehl-Neelsen stain. (C) Lowenstein-Jensen medium showing positive *Mycobacterium tuberculosis* culture growth.

**Figure 2:**
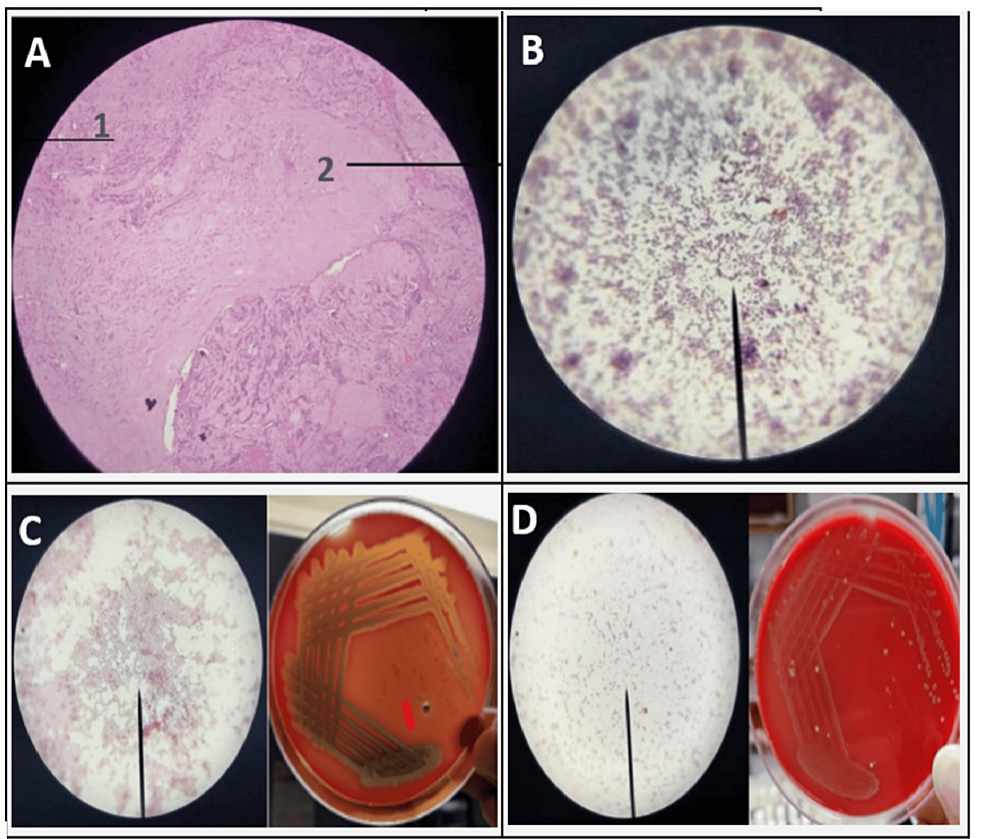
Pyogenic spondylodiscitis (A) Histopathology image of a case of pyogenic SpD showing neutrophillic infiltrate (labelled1) and tissue necrosis (labelled 2). (B) Light microscopy image of *Staphylococcus aureus* in clusters. (C) Light microscopy and MacConkey agar growth of *Pseudomonas aeruginosa.* (D) Light microscopy and MacConkey agar growth of *Salmonella typhimurium.*

Tubercular SpD was diagnosed in 21 (61.8%) patients, with culture positivity seen in 12 patients (Figure 1C), and GeneXpert positivity for tubercle bacilli was seen in 17 patients with no patient showing rifampicin resistance. Seven (20.6%) patients were positive in aerobic culture; etiologic organisms grown were *Staphylococcus aureus* (n=3) (Figure 2B), *Pseudomonas aeruginosa* (n=3) (Figure 2C), and *Salmonella typhimurium *(n=1) (Figure 2D). Overall combined MB positive results were seen in 28 (82.3%) patients.

HPE was positive in 24 patients with neutrophilic infiltrate suggestive of pyogenic SpD in six cases (Figure 2A) and caseating granulomatous inflammation suggestive of tubercular SpD in 18 cases (Figure 1A). HPE was non-specific in 10 of the 34 cases studied.

Final etiologic diagnoses

The final diagnosis after both HPE and MB was tubercular SpD in 62% (n=21) subjects and pyogenic SpD in seven (20%) patients. The subgroup in which both MB and HPE tests were inconclusive (18%, n=6) was categorized as Non-specific SpD (Tables 1, 2).

**Table 1:** Final etiological diagnosis (N=34)

Etiologic Sub-Groups		Number of Patients	Percentage
Final Diagnosis	Tubercular	21	61.8%
	Pyogenic	7	20.6%
	Non-specific	6	17.6%

**Table 2:** Combined utility of microbiology and histopathology in spondylodiscitis diagnosis

		Histopathology Reports				Total (N=34)
		Specific (N=24)		Non-specific (N=10)		
		Number of patients	Percentage	Number of patients	Percentage	
Microbiology diagnosis	Positive	20	83.3%	4	40%	24
	Negative	4	16.7%	6	60%	10

## Discussion

SpD is an osteomyelitis variant associated with significant morbidity and mortality that remains a challenging clinical entity worldwide despite therapeutic and diagnostic advances [5]. The most common symptom of 'backache', the absence of specific signs, a low index of suspicion, and non-contributory spine radiographs often cause late diagnosis. Its progressive course results in physical deformities and neurological deficits, which contribute to high morbidity. Pain relief, spinal stability, and prevention of neurological deficits are prime management goals.

Infective SpD is curable with appropriate antimicrobial therapy and, keeping with the current trend of evidence-based medicine, it is universally accepted that all forms of empiric therapy should be withheld till a definitive etiologic diagnosis is reached. However, in the specific instance of SpD, with its vague clinical profile, absence of diagnostic clinical signs, and relative inaccessibility of anatomic site, the onus of guiding therapy would rest on imaging modalities (CT, MRI, PET) to accurately identify the site, extent, and ideally the etiologic agent. MRI has proven itself as the imaging modality of choice in objectively demonstrating evidence of SpD and its complications but has low specificity in the identification of causative organisms. MB and HPE hold the potential of establishing the etiologic diagnosis and are ideal for targeted therapy, but have limitations of being time-intensive and dependent on tissue sampling adequacy. Further, a non-specific HPE and MB result may cause diagnostic uncertainty and delay the institution of definitive treatment.

The current study revealed more male patients affected by SpD in a male-to-female ratio of 1.5:1. All patients had back pain at presentation, with fever and constitutional symptoms being present in 25-30% of the participants, and neurologic deficits manifested only in 10-12%. Though an infective process, a relatively low incidence of fever was noted in our patients, similar to the incidence reported by other studies worldwide [6,7]. Predominant symptoms in tuberculous SpD (n=21) (after backache) were fever (11/21) and constitutional symptoms, which included weight loss, fatigue, and loss of appetite (10/21). Night pains were seen in one-third of the patients in the sub-group and spinal deformity was seen in two of the 21 patients with tubercular SpD. The pyogenic group of patients had predominant symptoms of backache and fever with a non-specific SpD sub-group with radicular pain as the second most common presenting symptom, raising the possibility of early SpD being misdiagnosed as degenerative etiology. 

In the preliminary laboratory profile, only ESR was raised in >90% of patients, whereas CRP level and WBC counts were raised in only 17% of patients, which was identical to findings reported by Yasar et al. [8], but contrary to findings reported in certain studies [1,9,10], where elevated CRP was associated with SpD. Leucocytosis was noted consistently only in pyogenic SpD. Though limited by low patient numbers, a combination of elevated ESR with leucocytosis in suspected patients of SpD would favor pyogenic etiology as per our study.

As reported widely in literature, the dorsal and lumbar spine accounted for about 80% of cases in our series. Tuberculous SpD was common in the dorsal spine while lumbar spine was the most common site for pyogenic SpD, which corresponds to vertebral levels reported in studies by Frel et al. [10]. The role of MRI Spine as the primary imaging modality was re-affirmed in the current study; however considerable overlap in MRI findings was noted between the three subgroups, limiting the discriminatory role of MRI. This is concurrent with the literature available [11], stating difficulty in diagnosis purely based on imaging. 

All 34 patients underwent tissue sampling, with open biopsy in 29 patients and CT-guided needle biopsy in five. Most patients who reported to our hospital presented in advanced stages of the disease with progressive neurologic deficits/ deformity, spinal instability, persistent (or recurrent) positive blood cultures, or worsening pain, mandating surgical intervention. On the basis of the sampling technique utilized, it was found that in the 29 patients who underwent open biopsy, conclusive MB findings were noted in 86.2% (24/29) of patients, and 72.5% (21/29) were proven HPE positive. Of the patients who underwent CT-guided biopsy, there was an identical 60% positive outcome noted (3/5) on both diagnostic tests. These numbers were higher than previous studies, as seen in the meta-analysis by McNamara et al. [12], who found that tissue samples collected from surgical sites often yielded the best results of up to 76% only, while in a study by Pandita et al., CT-guided biopsy had positive culture rate of only 18% and subsequent open biopsy yielded positive culture in only 50% [9].

MB had culture positivity in 19 patients (56%) in the current study, including culture-positive pyogenic samples in seven patients and positive growth for AFB in 12 patients. GeneXpert for tuberculosis was positive in 17 patients, which included all 12 patients with positive AFB culture and five patients with negative AFB culture; therefore the net combined conclusive results for MB methods in the current study was in 24 out of 34 patients (70.6%).

HPE was also conclusive in 24 patients (70.6%), with definitive pointers towards tubercular etiology in 18 patients (53%) and pyogenic SpD in six patients (17.6%), with 10 tissue samples (29.4%) reported as ‘Non-specific’. Out of this non-specific HPE sub-group, three patients had MB evidence of tubercular etiology (in smear, culture, or GeneXpert) and one patient was diagnosed as pyogenic SpD on the basis of aerobic culture of *Salmonella typhimurium*.

In our study, in 24 of the 34 patients, a definitive diagnosis could be arrived at using only HPE or MiB as the diagnostic gold standard. But significantly, 10 out of 34 studies were inconclusive and hence underwent delay in treatment, repeat biopsy, or empirical therapy. However, in this subgroup, there were four patients in whom the positive spectra overlapped, and etiologic diagnosis could be arrived at on the basis of either MB or HPE being positive. Hence, combining findings of HPE and MB, augmented the probability of reaching a definitive diagnosis as shown in Table 3*.*

**Table 3:** Sub-groups of tuberculous spondylodiscitis based on diagnostic techniques "+" denotes positive results; "-" denotes negative results NS: non specific; AFB: acid-fast bacillus; HPE: histopathology

Sub-Group	AFB culture	Gene Xpert	HPE	Number
I	+	+	+	9
II	-	-	+	4
III	+	+	NS	3
IV	-	+	+	5

The subset reported as 'Non-specific' SpD in the present study is what raises the pathophysiologic, diagnostic, and management dilemma, hence representing the most challenging entity. In our opinion, these patients could represent sub-acute/chronic infective process compounded by intermittent courses of empiric medical therapy with broad-spectrum anti-microbial/anti-inflammatory agents, as was also proposed by Kamal et al. [13] in their observations in Egyptian patients. Uncommon etiologic organisms that escape the conventional MB culture radar to remain undetected, as suggested by sporadic case reports of rare causative agents isolated from SpD biopsies worldwide [14-16], is another postulate for this phenomenon. Another interesting observation on this sub-group finally categorized as 'Non-specific SpD' is related to epidemiological features. The patients were predominantly women, with an average age above 60 years; this disparity can hypothetically be due to neglected disease and chronic progression in elderly female patients, compounded by the coronavirus disease (COVID-19) scenario.

This study showed that SpD can exhibit assorted permutations and combinations of HPE and MB outcomes in a given patient, varying from positive reports for both tests which mutually support the etiologic diagnosis vis-a-vis those having negative reports for both tests, which does not rule out SpD and therefore creates a therapeutic dilemma. Less frequently, either one of the tests is reported positive, in which case, it is correlated with the clinical profile and accepted as diagnostic evidence to initiate targeted therapy. This phenomenon was clearly depicted in the four distinct sub-groups generated in patients diagnosed with tubercular SpD in our study, as shown in Table 3. 

Only nine of 21 (43%) had all culture, GeneXpert, and HPE findings reported as positive. In the remaining 57%, there was a nearly equal distribution of patients with only HPE positive, with only MB and GeneXpert positive (HPE non-contributory), and with GeneXpert and HPE positive (culture negative). Though not seen in our study, based on recent reports on the utility of GeneXpert [17,18], the potential fourth option would definitely be found in larger series in the future, where only GeneXpert studies would be positive.

In our institution, once a diagnosis was established, patients were started on either a standard course of anti-tubercular therapy (ATT) for tubercular SpD or culture-sensitive antibiotics IV for two weeks followed by oral antibiotics for four weeks for the pyogenic subgroup. Response to treatment was monitored based on clinical improvement and a decreasing trend in ESR and CRP values on follow-up. 

Of the six patients of the non-specific subgroup, four had undergone surgical intervention and open biopsy. The challenge in such patients is in decision-making, mainly with regard to performing a repeat biopsy, as has been suggested in some institutional series [19]. The caveat for a repeat biopsy is that the process is invasive and time-intensive, besides having a risk of repeat negative results. This limitation to the role of HPE and MB in SpD patients has also been highlighted by Bhagat et al. [20]^ ^and Tachibana et al. [21] in their analyses previously. 

The decision to start on empirical antibiotics without repeat MB and HPE, based on clinical judgment, should be indicated in critical conditions such as sepsis, extreme morbidity, or deteriorating general condition of the patient. For the remaining subset of non-specific SpD patients, empiric antibiotic therapy can be given under medical supervision to monitor response, and pro-active modification of antimicrobials if the disease process is observed to be progressive while on a treatment line.

## Conclusions

The current study has asserted the requirement of adopting a standard structured approach at the institutional level, to sequentially and systematically evaluate SpD patients. It should commence by having a high index of suspicion based on the presenting clinical profile, followed by early performance of MRI Spine followed by tissue sampling for HPE and MB to reach an etiologic conclusion and administer specific antimicrobial therapy.

In our limited experience, HPE is more sensitive in determining SpD as compared to traditional culture-staining MB techniques, but the addition of the modern GeneXpert technique as a supplement to culture has improved the MB positivity rate. Despite open biopsy done in the majority of patients in this study, tissue samples were inconclusive for etiology on both HPE and MB in a subset of patients. We, therefore, recommend that an empiric therapy regimen should be formalized within the Institutional Antibiotic Policy and reserved for the inevitable subset of patients finally diagnosed as ‘Non-specific SpD’ on having non-contributory HPE and MB reports.
